# Supplementary figures and images for: Independent component analysis recovers consistent regulatory signals from disparate datasets
Source: PLoS Comput Biol. 2021 Feb 2;17(2):e1008647. doi: 10.1371/journal.pcbi.1008647 (PMC7888660; doi:10.1371/journal.pcbi.1008647)

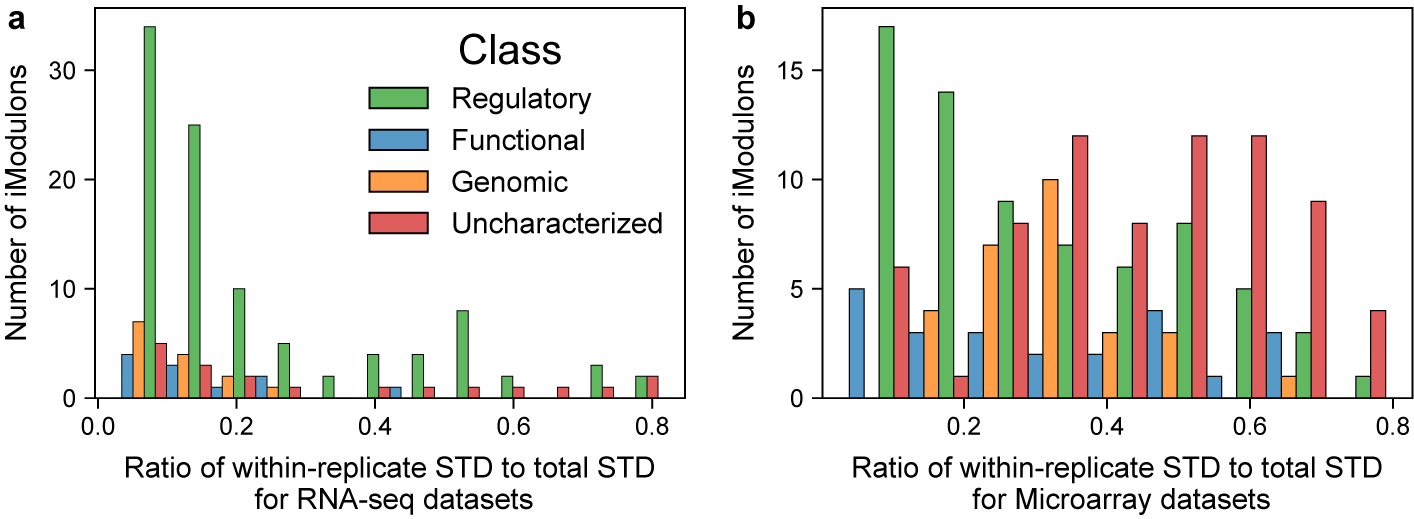

Supplement: S1 Fig — Relative standard deviation (STD) of iModulon activities between replicates for each iModulon in ​(a) RNA-seq datasets, and (b) microarray datasets. (TIF) [file pcbi.1008647.s001.tif]

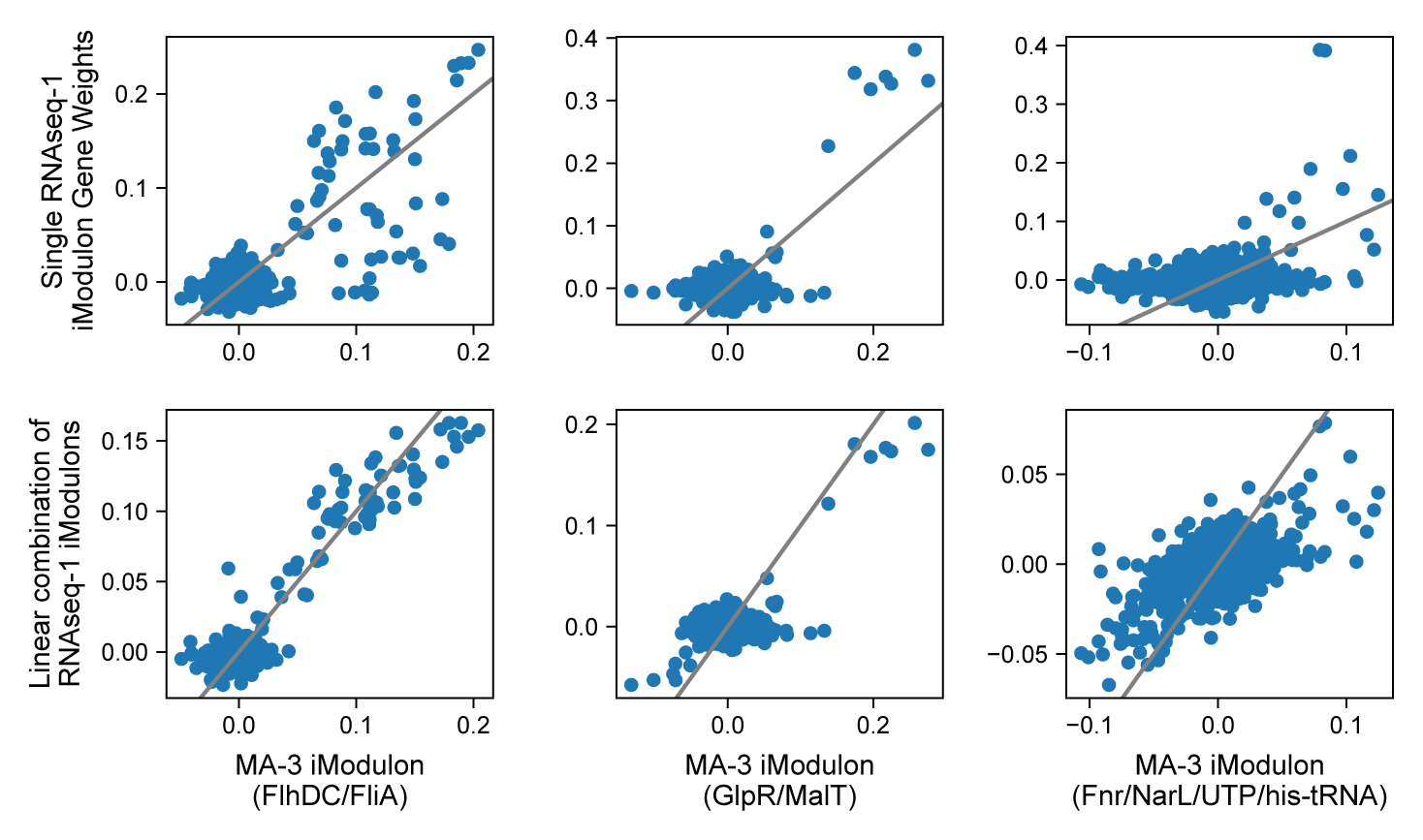

Supplement: S2 Fig — Top scatter plot shows the MA-3 iModulon gene weights compared against the best hit iModulon in the RNAseq-1 dataset. Bottom scatter plot shows the linear combination of RNAseq-1 iModulons listed on the x-axis label. (TIF) [file pcbi.1008647.s002.tif]

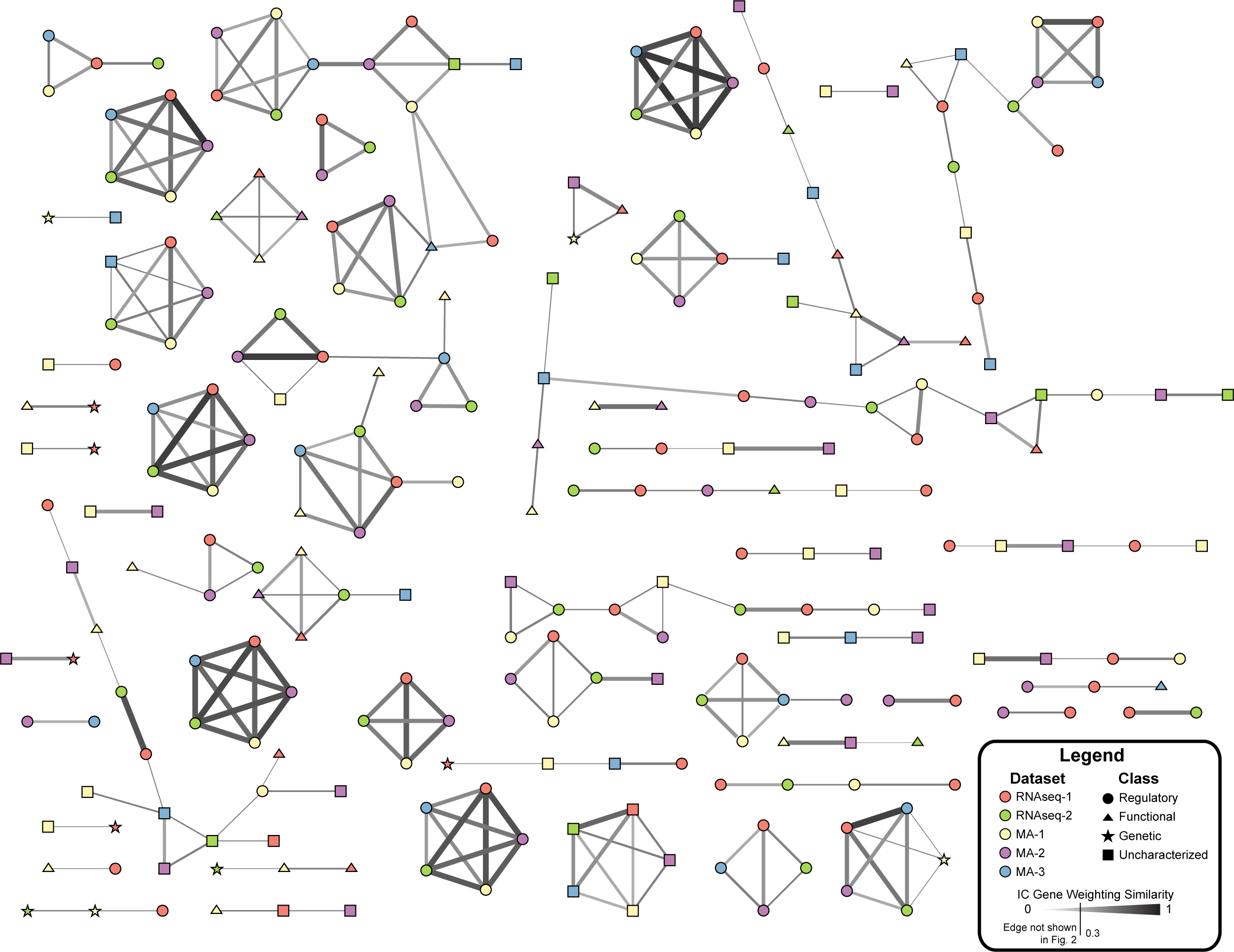

Supplement: S3 Fig — Edges with an IC Gene Weighting similarity score below 0.3 were pruned from Fig 2. (TIF) [file pcbi.1008647.s003.tif]

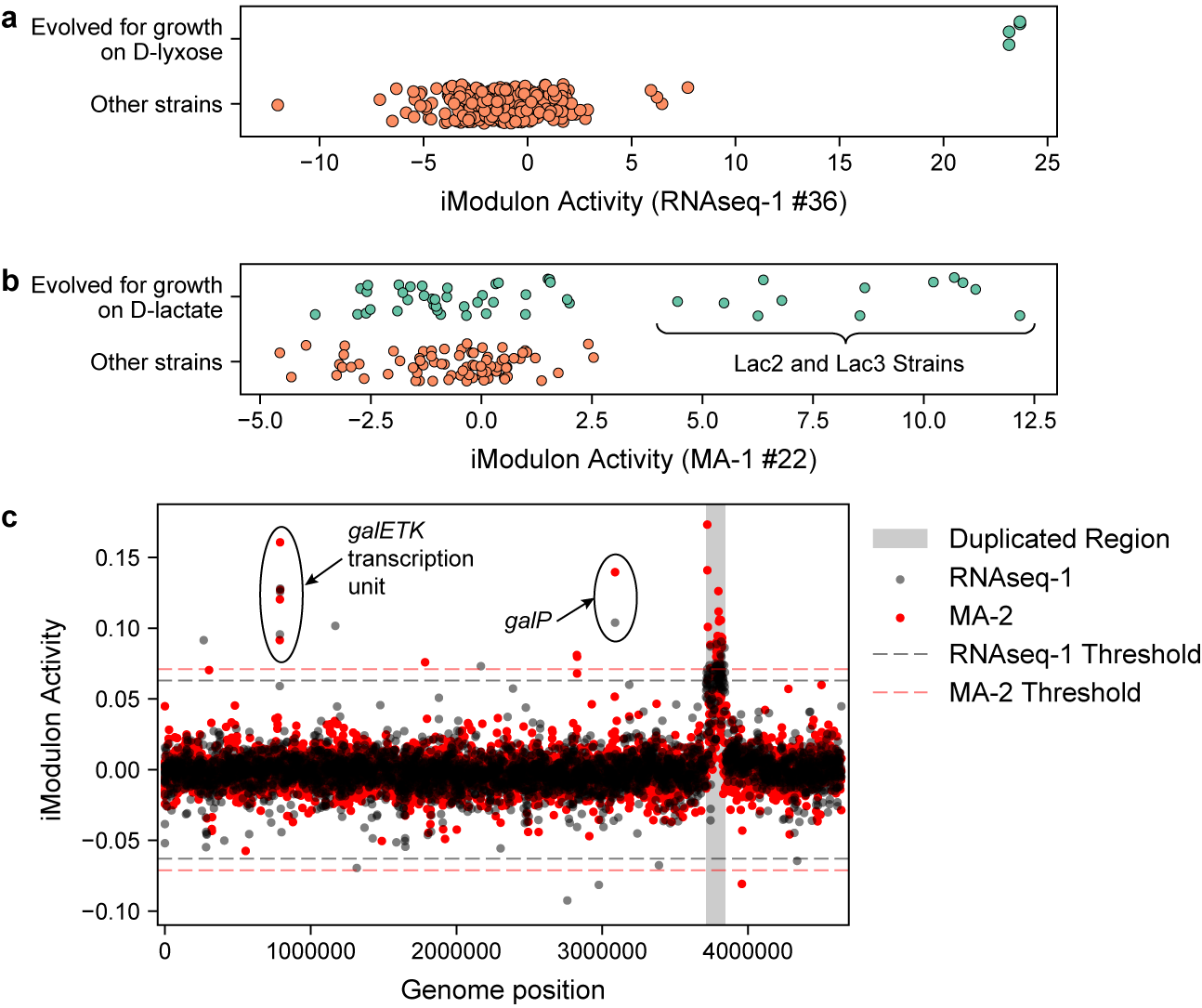

Supplement: S4 Fig — (a) Activities of the iModulon in the RNAseq-1 dataset separate E. coli strains evolved for growth on the non-native carbon source D-lyxose from the other strains in the compendium ​[1]. (b) Activities of the uncharacterized iModulon in MA-1 that was linked to the iModulon described in panel (a). Seven strains were evolved in parallel for growth on D-lactate ​[38]​, but only two endpoint strains (named Lac2 and Lac3) exhibited high iModulon activities. These strains were not re-sequenced, so the adaptive mutations could not be confirmed. (c) Scatter plot showing the IC gene weightings corresponding to the iModulons described in panel (a) (in black) and panel (b) (in red). Thresholds determining iModulon composition are indicated by dashed lines. The genomic duplication from the D-lyxose-evolved strains is highlighted in gray, indicating that all strains with high activities likely acquired an identical duplication along their evolutionary trajectory. Two transcription units outside of the duplicated region were captured in both iModulons, the ​galETK transcription unit, and the ​galP gene. These genes are responsible for D-galactose catabolism. (TIF) [file pcbi.1008647.s004.tif]

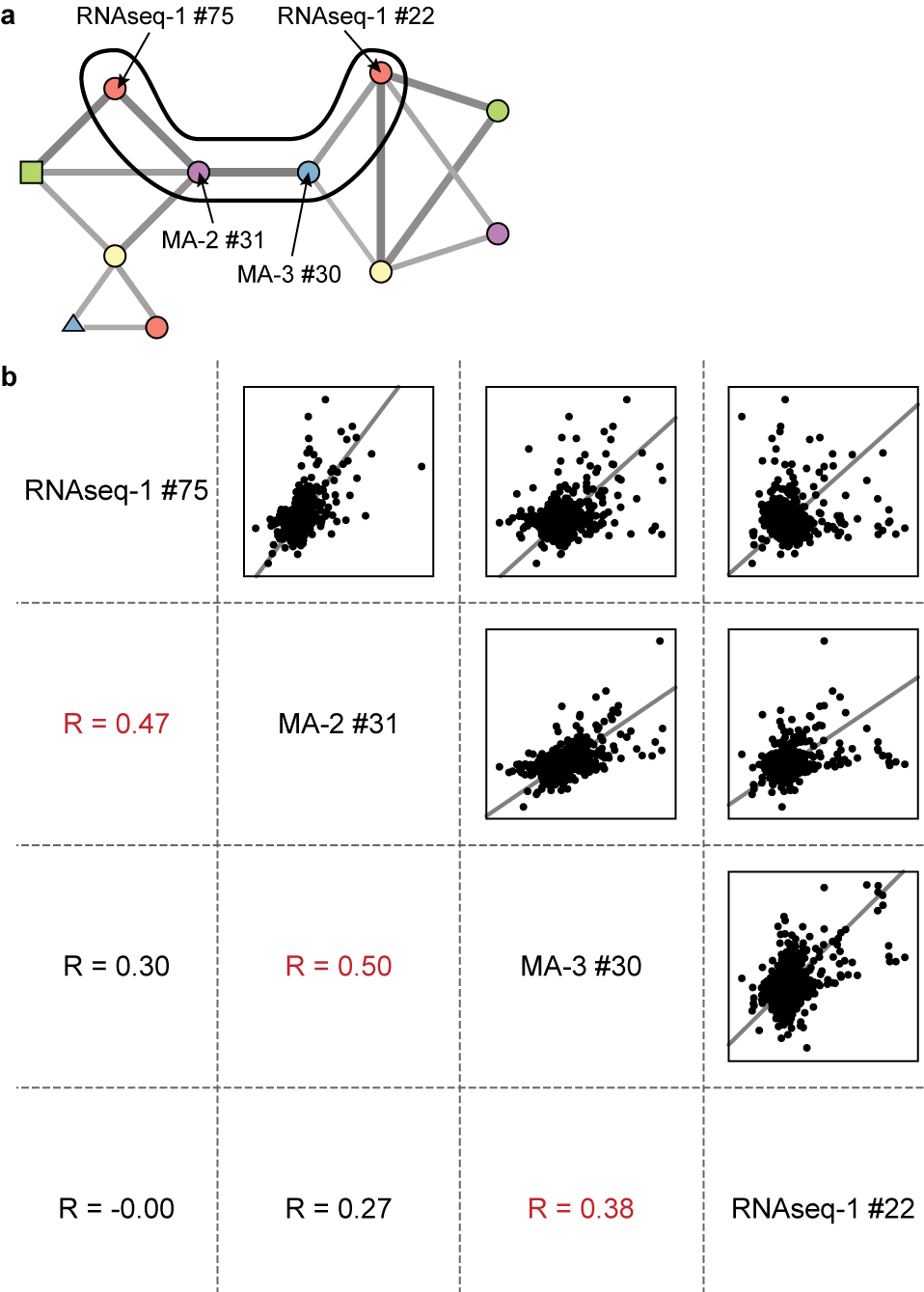

Supplement: S5 Fig — Each dataset contains a different set of conditions, which can activate different groups of respiration-related genes leading to different co-expression patterns between datasets. (a) The complex iModulon cluster shows that two iModulons from the RNAseq-1 dataset are indirectly connected. (b) Scatterplots of the IC gene weights for the iModulons highlighted in (a). The Pearson R correlation of the IC gene weights is shown below. The two RNAseq-1 iModulons show no correlation, but still contain a few genes in common, indicating that the expression of these shared genes are controlled by two distinct underlying sources. (TIF) [file pcbi.1008647.s005.tif]

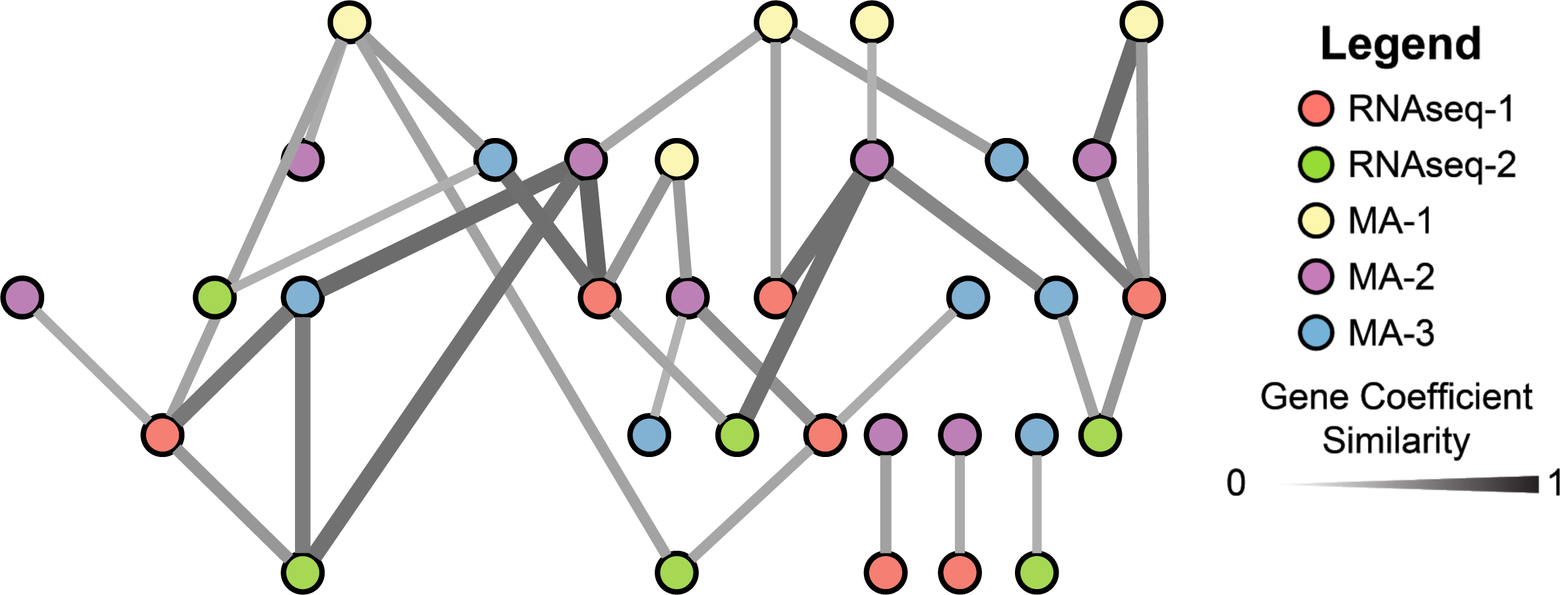

Supplement: S6 Fig — Edges with a gene weighting similarity score below 0.3 were pruned from this figure. Few iModulons have a reciprocal best hit, and no conserved clusters exist when analyzing Principal Components. (TIF) [file pcbi.1008647.s006.tif]

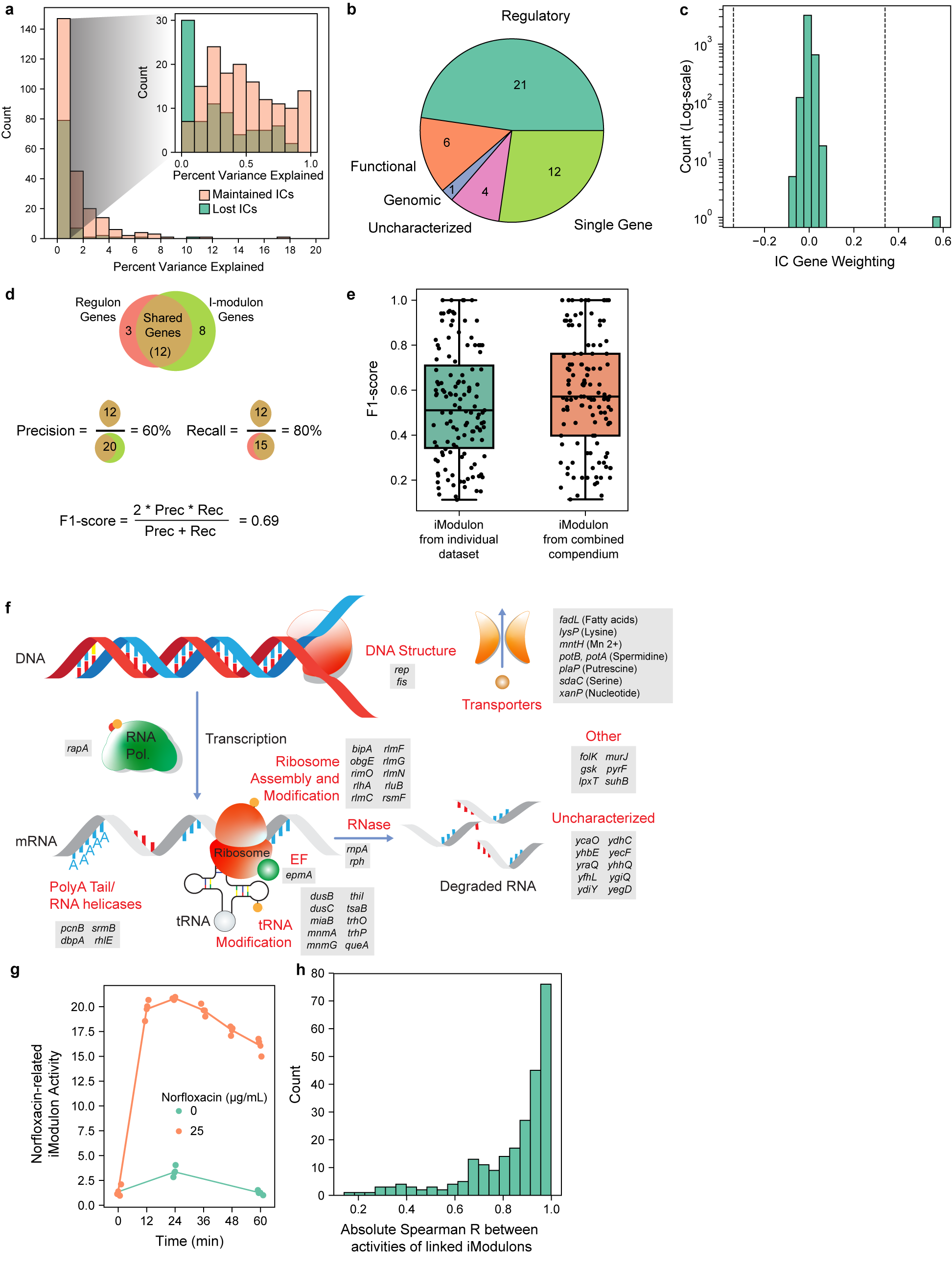

Supplement: S7 Fig — (a) Histogram of the percent of total expression explained by each independent component in the individual datasets. Components that are maintained (i.e., identify an RBH hit with the full compendium decomposition) are colored in pink, whereas components that are lost upon data integration are colored in blue. (b) Pie chart illustrating the classes of the new iModulons extracted from the combined datasets. (c) Histogram of the IC gene coefficients of a Single Gene component. Dashed lines indicate the iModulon threshold. (d) Schematic illustration of precision, recall, and F1-score. (e) Boxplots of the F1-scores between iModulons and their associated regulators for Regulatory iModulons. Only Regulatory iModulons in the individual dataset that found an RBH in the full compendium are shown in the left boxplot, and the RBH of these iModulons in the full dataset are shown in the right boxplot. ​(f) Schematic illustration of the various processes encoded by the genes in the Central Dogma iModulon. (g) Time-course treatment of DNA-damage inducing norfloxacin activates an iModulon that shows reduced activity when RNAP is bound by ppGpp. (h) Histogram of absolute Spearman correlations between iModulon activities in components that are RBHs in the full dataset compared to the individual datasets. (TIF) [file pcbi.1008647.s007.tif]

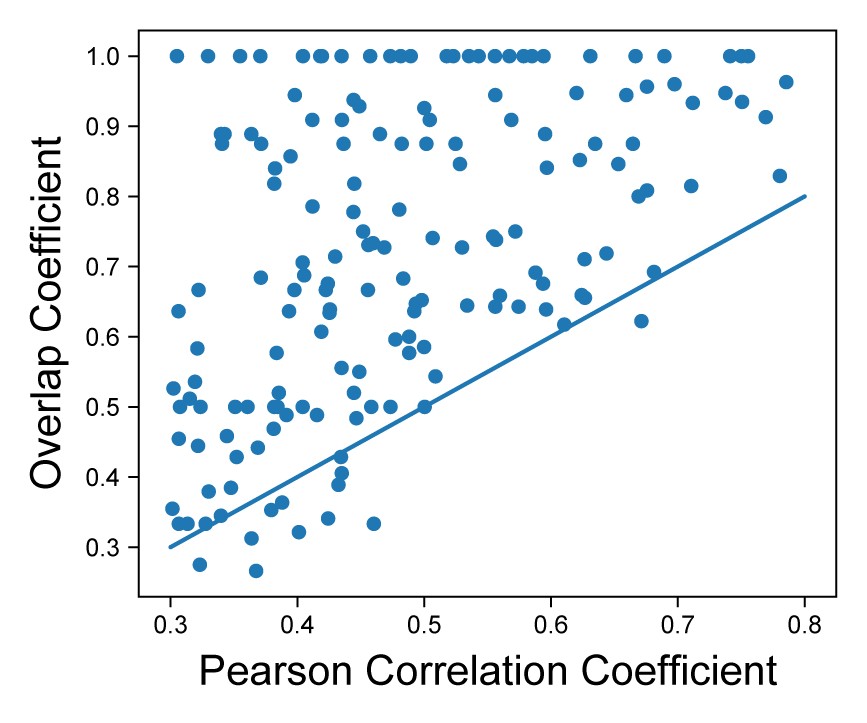

Supplement: S8 Fig — (TIF) [file pcbi.1008647.s008.tif]
