# Supplementary material for: Independent component analysis recovers consistent regulatory signals from disparate datasets
Source: PLoS Comput Biol. 2021 Feb 2;17(2):e1008647. doi: 10.1371/journal.pcbi.1008647 (PMC7888660; doi:10.1371/journal.pcbi.1008647)
Supplement: Attachment — Submitted filename: reviewer_responses.pdf [file pcbi.1008647.s020.pdf]

Dear Editor,

We would like to thank you and the reviewers for their constructive comments regarding the enclosed manuscript titled: “Independent component analysis recovers consistent regulatory signals from disparate datasets”. When reviewing the comments, we realized that some reorganization was necessary to clarify the novelty and scope of our manuscript. To this end, we have made the following structural changes to the manuscript:

1. We have changed the title of this manuscript to “Independent component analysis recovers consistent regulatory signals from disparate datasets”.
2. We added a new section, titled “iModulon structure is consistent across genetically diverse strains”, that shows how iModulons can characterize *E. coli* strain variants. Specifically, this section shows how iModulons can clearly link mutations acquired during laboratory evolution to specific changes in gene expression.
3. We added another new section where we apply ICA to COLOMBOS, a dataset of over 3,000 transcriptomes. Through this analysis, we identified three iModulons that most likely represent undiscovered regulons, and propose biological functions for these regulons.
4. We removed the section discussing ICA of the proteome, as the results were seen as inconclusive.
5. We split the section previously titled “An iModulon controlled by CysB exists in all five datasets” into two sections for clarity.
6. We split the section previously titled “Data integration reveals increased resolution of iModulons” into three sections for clarity.

We hope that these changes enhance the novelty of our manuscript and clarify the results.

Editor comments:

Major comments:

1. There is no analysis/discussion regarding the differences that can (and most likely will) arise from grouping different strains together. Strains resulting from ALE can harbour substantial differences in regulatory program, and this effect can be much greater in natural variants (even the genome size can differ by >10%). I recommend to discuss this, whenever possible with supporting additional analysis.

In order to address this issue, we have included a new section titled “iModulon structure is consistent across genetically diverse strains” that outlines the various effects that ALE mutations can have on the regulatory program. We note that most of the regulatory

re-wiring seen in ALE experiments does not affect the iModulon structure, but rather modulates the overall activity of an iModulon, as shown in the selected examples.

2. Given that future application for the method is going to be either much larger datasets or other species (as authors point out), I think the manuscript would much benefit by expanding the scope in this direction. This could be done, e.g., by analysing datasets for additional species of health/industrial importance, and/or by including cross-species comparison, and/or by increasing the number of datasets. This would also help addressing the questions of novelty raised by the other reviewer.

Thank you for the suggestion. We have massively scaled up the analysis to include over 3,000 datasets from the COLOMBOS microarray compendium. The results are described in a new section titled “Predicting new regulons using big data”. We identify three iModulons that are highly likely to represent regulons, including one that is responsive to antimicrobial agents such as polymyxin B and colicin.

Minor comments:

1. Fig 2: X-axis labels unclear. R would be better indicated inside the plot and not as a x-axis label.

We have re-organized the axis labels in Figure 2, and moved the R values inside of the plot.

2. Table 1: Please consider removing the column ‘Research Group’. This is redundant with the next column (reference) and also unnecessarily gives an impression of subjectivity.

Thank you for the suggestion. We have removed the column from the Table.

Reviewer #1:

The paper by Sastry et al presents some results based on the use of ICA to recover regulatory signals from different datasets. The topic is a relevant one, and overall the study presented is, in my opinion, technically sound and presents some technical details useful for the community. Still, I believe that the results of the paper do not represent a major breakthrough to warrant its publication in PLOS CB.

Indeed, the main contribution of the paper over previous work is, as stated in the last paragraph of the Introduction, to show that the method provided in previous work based on ICA is capable of retrieving consistent regulatory modules from different transcriptomics and proteomics datasets for E. coli. Although some results point to consistent modules, I believe that the work shown here is still preliminary, showing somewhat marginal contributions over previous work, and some of the claims need more support. Some reasons for this statement are listed below:

- the analysis of the consistency of the iModulons obtained in based on Pearson correlation of the coefficients of the ICA (a metric of linear correlation while ICA can capture nonlinear relationships; maybe mutual information would be an option);

The major finding in this manuscript was that many iModulons exist in multiple datasets from different platforms, and these iModulons have linearly correlated, and often equivalent, gene weights (as shown in Figure 2a). The linear correlation is an important aspect, because it shows that the iModulons affect genes by similar factors across different datasets. For example, CysD is one of the top 3 genes in all CysB iModulons, which implies that its expression level changes substantially when the CysB regulatory activity changes. We felt that this phenomenon was best represented by a Pearson correlation coefficient.

However, we have also tested other non-linear correlation metrics, such as Spearman R and mutual information. The Spearman R correlation produced a graph with nearly identical connectivity, whereas mutual information produced a graph with fewer highly-reproducible iModulons and more complex iModulon connectivity (as defined in Figure 3a). Since Pearson R correlations (1) represent linearly correlated gene weights, (2) are easier to interpret, and (3) produce cleaner results, we decided to remain with the Pearson R correlation coefficient.

- a match of different modules in different datasets is obtained based on reciprocal best hit; no analysis is provided on the number of shared genes. Also, RBH is done pairwise and thus the 45% of linked iModulons is an interesting result, but not of a level that justifies some of the produced claims. The number of modules consistent in all 5 datasets (or even 3-4) seems to be low (even the example presented of CysB only holds for 4 datasets), although this is not discussed.

We have added another supplementary Figure (Figure S8) that shows that the Pearson R correlation coefficient between iModulons is close to the lower bound for the overlap coefficient (Szymkiewicz-Simpson coefficient), which compares the overlap between two finite sets. We also included the following statement in the Methods:

> “The Pearson correlation between two iModulons serves as a lower bound of the Szymkiewicz-Simpson overlap coefficient, which measures the level of overlap between two differently-sized sets.”

As for the number of highly-reproducible iModulons, we actually do not expect to see a significant fraction of iModulons appearing in all five datasets. This is because an iModulon can only be detected if the genes in the iModulon have expression changes in the dataset. This is dependent on the experimental conditions that are profiled in each dataset, which are highly varied between each of the five datasets. This is one of the major differences between our work and previous investigations into matrix decomposition reproducibility. We discuss this in the fourth paragraph of the discussion section.

In addition, the RBH method introduces an artificial restriction where each iModulon is linked to only one iModulon in another dataset. However, we have shown in the CysB section that iModulons in small datasets may consist of a linear combination of iModulons from larger datasets. We added the following text in the manuscript to help clarify:

> "An additional nine iModulons were found in three or four datasets, forming moderately-reproducible clusters. The missing iModulons could either be explained by low variation of iModulon genes in the missing dataset, or by many-to-one linkages between iModulons that could not be captured by the RBH dataset, as demonstrated with the MA-3 iModulon (See Figure 2b and Supplementary Figure 2)."

- the RBH by itself does not represent a high consistency between the connected modules, only that they are the best candidates to be connected.

This is true, which is why we also required correlation coefficients to be above R = 0.3 for the RBHs. We included Supplementary Figure 3, to show the RBH graph if we did not prune low correlation edges.

- it is not obvious why almost half of the modules retrieved from microarrays datasets cannot be characterised.

We investigated this further, and found that the activities of uncharacterized iModulons tend to have high variability between replicates. This effect is amplified in the microarray datasets, and less visible in the RNA-seq datasets. Therefore, we believe that technical variability may be captured in the microarray iModulons, leading to a high number of uncharacterized iModulons.

We have added Supplementary Figure 1 and the following text in the main manuscript to summarize this analysis:

> "To investigate this difference, we computed the relative variance of iModulon activities between replicates for each iModulon in all five datasets (Supplementary Figure 1). Uncharacterized iModulons have significantly higher variance between replicates than characterized iModulons (Student's t-test p-value < 10^{-10}), indicating that Uncharacterized iModulons tend to capture expression heterogeneity between replicates.
>
> In summary, application of ICA to expression datasets identifies biologically-relevant iModulons in both microarray and RNA-seq datasets. However, microarray iModulons are more likely to represent technical noise in the dataset."

- the results shown for the proteomics datasets seem to be very inconclusive and could be better discussed; they do not seem to justify the initial claim (in the abstract: "echoes of this structure remain in the proteome, accelerating biological discovery through multi-omics analysis"); also, the final sentence of that section is rather confusing.

We agree with this sentiment and have removed the proteomics results from this manuscript. We plan to generate additional proteomics datasets in the near future that will improve this analysis.

- comparisons with known regulators and regulated genes are only done via statistical tests/ enrichment and it would be interesting to compare possible composition of extracted modules as sets of genes. It would thus be interesting to see if this method could capture regulatory modules that could be used for predictive tasks.

Thank you for this suggestion. We have incorporated this idea into a new section entitled "Predicting new regulons using big data", where we predict three new regulons and provide hints towards their functions.

- the code in github is only partial; the full reproducibility of the results, although possible in theory is made harder since the scripts run to generate the results are not made available.

We have created a new github repository that walks through every step of the analysis at https://github.com/SBRG/xplatform_ica_paper. This includes Jupyter notebooks designed to walk anyone through the creation of each figure in the paper.

Minor comments

- in the Introduction (3rd paragraph; sentence starting with "Previously, ..." it is not clear that when describing previous work that it comes from ref 36, not cited there; given the importance of this previous work, it should be more clear.

Thank you, we have added the citation.

Finally, I note that since the study works over the same organism, it is "known" that the regulatory modules should be shared (while we may not know all of them) and are in fact underlying the generation of all these datasets. So, it would be surprising to find that the modules retrieved from these datasets would not contain some similarities. So, in a way, what would be surprising would be the lack of this consistency.

This was our initial hypothesis; that regulatory iModulons are shared across datasets regardless of expression profiling platform. However, there have not been any studies previously that have rigorously tested or proven this hypothesis. The only similar study we have found was the Cantini et al 2019 paper in Bioinformatics, which mainly assessed reproducibility of components across datasets in the same microarray platform, rather than across multiple platforms.

To enhance the impact of this study, we have added two new sections. One focuses on biological re-interpretation of laboratory evolved strains to antibiotics, and the second expands our results to a large, pre-processed microarray dataset containing over 3000 samples. We hope that these new sections will provide novelty by (1) mapping mutations to changes in transcription factor activities, and (2) predicting three new regulons.

Reviewer #2:

Summary of the paper:
The authors have used five independently produced datasets of RNAseq and microarray, and performed an ICA analysis on them to find underlying consistent patterns in latent space. Each independent component consists of weights of genes, and the few genes with a significant weight are grouped as iModulons. The iModulons were then characterized as regulatory, functional, genomic, or uncharacterized. Some of these iModulons were found in all datasets, and many were found in at least two, indicating that this technique can find relevant features of genetic data.

Major Comments:

My main comment is novelty, the authors use same technique/same philosophy published recently (ref 26) and similar research design, i.e. multiple cancer datasets comparison, ref 35, so message Matrix factorization recovers consistent regulatory signals from disparate datasets is not new.

There are a few differences between our manuscript and reference 26 that we would like to highlight. First, we compare 5 datasets with completely different experimental conditions and that use three different platforms (two microarray chips and RNA-seq), whereas the previous reference only compares homogeneous datasets using the same microarray platform. Second, we show that the iModulon structure is conserved after integrating disparate datasets, which was not addressed in the previous study. This is an important aspect of this study, since large datasets from a single source are difficult to create. Our findings show that we do not need data from a single study or research group to extract co-regulated gene sets. Instead, data from a variety of sources can be compiled into a large compendium and analyzed as a whole.

I would recommend to authors focusing on biological story, otherwise from low-rank matrices is pretty clear that they are the similar between biological experiments, especially when dealing with linear techniques such as ICA.

Thank you for the suggestion. We have added a new section focusing on the biological applications of this method to compare strains evolved to tolerate antibiotics. In addition, we have added another new section predicting three new regulons based on iModulons, and propose putative characterization of the regulons.

We would also like to point out that most linear techniques do not show this level of reproducibility. For example, our analysis of principal components (PCs) shows that only a select few PCs are reproducible across datasets, whereas most PCs are unique to specific datasets (Figure S6). To emphasize this point, we have changed the manuscript title to “Independent component analysis recovers consistent regulatory signals from disparate datasets”

Also although authors did analysis on log-transformed data, I would like to see it this analysis holds true on standardized data (mean-centered and scaled to unit variance), otherwise ICA is sensitive to scale of data and genes that are highly expressed in one dataset are also same genes that are highly expressed in another.

As for the data transformations, we actually centered each dataset to a reference condition, as to remove any bias towards or against genes that are constitutively highly expressed. This is similar to mean-centering, except the reference condition will not change if we append new expression profiles to a dataset.

We normally do not scale gene expression to unit variance, as this would inflate the effect of expression noise. For example, the variability of a gene whose expression that ranges 5-fold (from 5 log-TPM to 10 log-TPM) should be weighted more than a gene whose expression does not change within 2-fold (i.e. varies from 5 to 5.05 log-TPM)

Otherwise, technically paper sounds well and nicely written with nice graphics.

Minor comments

I recommend to change short title “iModulons are conserved across disparate datasets” - iModulons is not something established, neither informative.

We have changed the short title to: Expression signals are conserved across disparate datasets
